# Rates and risks for prolonged grief disorder in a sample of orphaned and widowed genocide survivors

**DOI:** 10.1186/1471-244X-10-55

**Published:** 2010-07-06

**Authors:** Susanne Schaal, Nadja Jacob, Jean-Pierre Dusingizemungu, Thomas Elbert

**Affiliations:** 1 Department of Psychology, University of Konstanz, 78457 Konstanz, Germany; 2 Vivo Foundation, 78476 Allensbach, Germany; 3 Department of Psychology, University of Butare, Butare, Rwanda

## Abstract

**Background:**

The concept of Prolonged Grief Disorder (PGD) has been defined in recent years by Prigerson and co-workers, who have developed and empirically tested consensus and diagnostic criteria for PGD. Using these most recent criteria defining PGD, the aim of this study was to determine rates of and risks for PGD in survivors of the 1994 Rwandan genocide who had lost a parent and/or the husband before, during or after the 1994 events.

**Methods:**

The PG-13 was administered to 206 orphans or half orphans and to 194 widows. A regression analysis was carried out to examine risk factors of PGD.

**Results:**

8.0% (*n *= 32) of the sample met criteria for PGD with an average of 12 years post-loss. All but one person had faced multiple losses and the majority indicated that their grief-related loss was due to violent death (70%). Grief was predicted mainly by time since the loss, by the violent nature of the loss, the severity of symptoms of posttraumatic stress disorder (PTSD) and the importance given to religious/spiritual beliefs. By contrast, gender, age at the time of bereavement, bereavement status (widow versus orphan), the number of different types of losses reported and participation in the funeral ceremony did not impact the severity of prolonged grief reactions.

**Conclusions:**

A significant portion of the interviewed sample continues to experience grief over interpersonal losses and unresolved grief may endure over time if not addressed by clinical intervention. Severity of grief reactions may be associated with a set of distinct risk factors. Subjects who lose someone through violent death seem to be at special risk as they have to deal with the loss experience as such and the traumatic aspects of the loss. Symptoms of PTSD may hinder the completion of the mourning process. Religious beliefs may facilitate the mourning process and help to find meaning in the loss. These aspects need to be considered in the treatment of PGD.

## Background

The loss of a loved one through death is among life's most stressful experiences. Even though the death of a significant other can be a very painful experience, most bereaved persons return to an adaptive level of functioning after the loss and bereavement-related distress diminishes over time. In the past decade, there has been interest in those cases that fail to recover and become fully functioning again. Whether or not such prolonged and disabling grief should be listed as a separate diagnostic entity in DSM V is an ongoing debate. A prominent recent proposal to specify symptoms of pathological grief and to define diagnostic criteria stems from Prigerson and coworkers [1], who have developed and empirically tested consensus, diagnostic criteria for a new DSM Axis I disorder called Prolonged Grief Disorder (PGD). A diagnosis of PGD can be made if following the death of a significant other clients endorse at least one separation distress symptom (longing for the deceased or intense pangs of separation distress) and at least five of the following nine cognitive, emotional and behavioral symptoms, experienced daily or to a distressing degree: feeling emotionally numb, feeling stunned or shocked, feeling that life is meaningless, confusion about one's role in life or diminished sense of self, mistrust of others, difficulty accepting the loss, avoidance of the reality of the loss, bitterness over the loss, and difficulty moving on with life. In addition, symptoms must endure at least six months and be associated with significant functional impairment.

The study of epidemiology of prolonged grief reactions and the comparison of findings across studies have been limited by the absence of universally accepted, standardized criteria for diagnosis. Investigators use different criteria for grief outcomes, which makes the assimilation of results across studies difficult.

The first aim of the present study was to determine the rate of PGD using the recently proposed diagnostic criteria among orphaned and widowed survivors of the Rwandan genocide. Rwandans have suffered tremendous personal losses during the genocide in 1994. Over a period of 100 days more than 10% of Rwanda's eight million inhabitants were murdered. Previous studies have documented the wide range of traumatic events including losses suffered by Rwandan survivors [2,3]. A recent survey of 2,091 Rwandan adults documented that the majority (70.9%) reported having lost a close family member during the genocide [4]. Many orphans, half-orphans and widows are left behind by the genocide, and more recently by AIDS-related deaths. Whereas previous studies have reported on the incidence of depression and posttraumatic stress disorder (PTSD) among genocide survivors [3-6], to our knowledge, only one study has investigated the rate of prolonged grief reactions in Rwandan widows who had lost their husband during the genocide [7]. An estimated of 12.5% met the newly proposed diagnostic criteria for PGD. The present study aimed to replicate the findings in a larger and more heterogeneous sample, including orphans and widows who have been bereaved for different reasons.

A second goal of the present study was to investigate correlates and thus potential predictors of prolonged grief reactions. We aimed to examine individual factors (gender, bereavement status) and contextual and death-specific factors (mode of death, severity of symptoms of PTSD, time since the loss, number of types of losses, funeral attendance and importance of religious/spiritual beliefs).

In terms of demographic variables, gender and age are inconsistently reported risk factors in the development of prolonged grief reactions. Whereas some studies found that gender [8-10] and age [8,9,11] are predictors for the development of grief reactions, other authors documented no associations between grief and the demographic variables of gender [12-14] or age [9,10,13,14].

Several studies have shown that the loss of a spouse might result in more intense grief reactions than any other type of loss [9,12,13]. According to Morgan et al. [15], the death of a spouse also brings along other painful slumps in life: many have difficulties managing their household on their own or maintaining involvement in the lives of their children; and some are left with enormous financial difficulties and they may lack knowledge or skills in the areas for which their partner was responsible. In Rwanda remarriage is not socially tolerated. The chance of having more children who would provide in old age is therefore limited. Often the parent alive cared for half-orphans or they could live like many full orphans within their extended families. In contrast, widows mostly had to manage their lives on their own.

It has often been argued that the mode of death plays an important role in the development of prolonged grief. A number of studies have reported that the violent nature of the death constitutes a significant risk factor for the development of PGD [12,13]. Other studies have documented that violent deaths do not pose a heightened PGD-risk [for example 9]. The high rates of past violence in Rwanda allow us to compare grief reactions between bereaved survivors of violent and non-violent deaths. It is possible that the death due to extreme acts of violence might put additional strain on the normal course of grief because of the traumatic stress caused by the loss.

A large amount of studies have investigated the time that has passed since the bereavement as a potential predictor for grief symptom severity. However, most studies have sampled bereaved persons with no substantial variability in time since the loss and found no significant association between time since the death and the severity of prolonged grief symptoms or PGD diagnosis [9,12,16,17]. In the present study, where the time since the loss is expected to show a great variability, we examined if the time since the loss would be significantly associated with symptoms of prolonged grief disorder.

The term "bereavement overload" has been introduced into the grief literature to describe a phenomenon in which an individual confronts multiple losses, such that one loss cannot be accommodated before another occurs [18]. We examined if the number of types of losses would be a significant predictor of grief severity.

Funeral rituals might facilitate grief adjustment and might be particularly important in those cases in which death was not expected [19]. Other researchers have reported that the participation in a funeral ceremony had no effect on the grieving process [20,21]. In the context of the genocide, very often survivors might have not been able to participate in funeral services either because no funeral ritual could take place as bodies might not have been retrievable or the survivor was not able to participate due to ongoing threats to his/her life. We explored if funeral participation would facilitate the grieving process and entail less prolonged grief symptoms compared to those survivors who were not present at the funeral.

Little is known about the coping strategy of religious/spiritual beliefs; e.g. the importance of religiosity in the actual life of the bereaved. There have been some studies that point to the positive effects of religious beliefs on bereavement [22,23].

The first aim of the study was to examine the rate of PGD in a sample of orphaned and widowed survivors of the genocide. As a second goal, we examined the following potential correlates of PGD: gender, age at the time of bereavement, bereavement status (widow versus orphan), mode of death (violent versus non violent), severity of symptoms of PTSD, passed time since the bereavement, number of reported types of losses, participation in a funeral ritual and importance of religious/spiritual beliefs.

## Methods

### Procedure

The study was conducted in Butare, Rwanda in August/September 2007. It was approved by the University of Konstanz Ethical Review Board and by Rwanda's National Institute of Statistics, Kigali. Eligible subjects were widows (female gender) and orphans (female and male gender) suggesting that a loss experience was a precondition for participation. Furthermore, subjects needed to be at least 18 years old at the time of the interview and had to experience the Rwandan genocide in 1994. Widows were participants who had lost their husbands and who were not remarried. Orphans were participants who had lost at least one parent and who were child survivors of the genocide that is not older than 31 years at the time of the interview. The Joint United Nations Programme on HIV and AIDS (UNAIDS), United Nations Children's Fund (UNICEF), and other groups define any child that has lost one parent as an orphan [24]. The study procedure and aims of the study were explained to all participants and signed written informed consent was obtained from all subjects. Diagnostic interviews were carried out by 15 Master level psychologists and psychology students (7 female and 8 male) from the National University of Butare, Rwanda. The various questionnaires were translated into Kinyarwanda and translated back by Master level psychology students from the University of Butare. Raters were trained during an intensive 2-week training by two female psychologists (S.S. and N.J.) in the basic theoretical concepts and in sensitive and empathic interviewing techniques. The first interviews in the field were conducted under the supervision of the psychologists and the interviewers received extensive feedback. Interviews were carried out in five of the following randomly selected sectors of Butare: Tumba, Mukura, Mbazi, Huye and Ngoma. Three trained raters were randomly assigned to each sector and in each sector three quarters were randomly selected (one quarter per person). Meetings were arranged every other day to supervise the quality of the interviews, to review the questionnaires and to provide feedback. The study was conceived as a community-based study with a house-to-house survey. Interviewers went house-to-house, starting at a convenient location within the assigned quarter. Each subsequent house was approached until the required number of interviews was achieved. Dwellers were asked if any widows or orphans resided within the home. If an orphan or widow was identified by the family, the interviewer then clarified if inclusion criteria were met. Houses were re-approached at a later time, if nobody was encountered or available at the first visit. If both a widow and an orphan were living in the same household, both were interviewed, if available and willing. If more than one orphan was living in a household, one was chosen randomly for participation. The interview lasted about two hours and was conducted in the respondent's home. After the interview, interviewees received 1000 Rwandan Francs (about 1.30 Euro) for their participation.

### Instruments

Socio-demographic information was obtained, including gender, age, educational background, monthly income of the household and various variables concerning religion (religious affiliation, importance of religious/spiritual beliefs, and number of weekly religious activities). We assessed the importance of religious/spiritual beliefs on a 4-point Likkert scale from 0 (not at all important) to 3 (very important) using the following item proposed by Brown et al. [25]: "In general, how important are religious or spiritual beliefs in your day-to-day life?" To assess religious behavior, participants were asked, "How often did you participate in religious activities in the past week?", measured by frequency of church attendance and private religious activities. Some death-specific questions were administered including the kind of losses ever experienced, the grief related loss (worst loss, indicating the loss which was personally experienced as the most disturbing and to which the prolonged grief reactions referred to), the mode of death of the worst loss, passed time in years since the worst loss and whether a funeral ceremony of the grief-related loss took place and whether the subject had attended this funeral service. If the most distressing loss had occurred during the genocide, it was ascertained whether the dead body had been retrieved. The number of types of losses was calculated by summing up the number of the different types of losses ever experienced including the loss of a partner, at least one child, the mother, the father, at least one sibling, at least one other family member and at least one other close person (possible range: 0-7). PGD (diagnostic status and symptom severity) was assessed using the PG-13 [26]. However, the intrusion item has been deleted by Prigerson in this questionnaire since it is supposed to give no additional information from yearning (personal communication with Prigerson, 08.03.2007). PTSD was assessed using the PTSD Symptom Scale-Interview (PSS-I) [27]. The PSS-I assesses the 17 DSM-IV symptom criteria for PTSD and refers to symptoms experienced in the previous month. Each of the items was answered on a 4-point scale ranging from 0 (not at all/only one time) to 3 (5 or more times per week/almost always). A PTSD severity-score (possible scores range from 0-51) was computed by summing all symptom scores. The PG-13 is a structured diagnostic interview that assesses 11 potential PGD symptoms in the previous month. Each of these items is answered on a 5-point scale ranging from 1 (never/not at all) to 5 (several times a day/severe) to represent increasing levels of symptom severity. A PGD diagnosis requires that 1 of the proposed 2 "separation distress" symptoms and 5 of the 9 proposed "cognitive, emotional and behavioral" symptoms receive a score of at least 4 (at least once a day or marked). The grief-score includes the sum of the score of each of the 11 grief symptoms and ranges from 11 to 55. The PG-13 covers all symptoms that have recently been proposed for inclusion in DSM-V and that have been described above [1].

### Statistical Analyses

Descriptive data are presented, expressed as frequencies (%), mean scores and standard deviations. Chi square analysis, Kruskal-Wallis-Test and independent samples t tests are used to analyze between-group differences. To investigate the association between PGD and different predictor variables, a linear regression was calculated for the grief score. The following independent variables were entered simultaneously into the analyses: gender (female versus male), age at the time of bereavement, bereavement status (widow versus orphan), violent death (grief-related loss due to genocide, accident or poisoning versus death due to age, illness or other non-violent deaths), severity of symptoms of PTSD (PTSD severity score), years since the loss, number of types of losses, participation in a funeral ritual and importance of religious/spiritual beliefs. We examined the correlations between the dependent and independent variables entered into the regression model using Phi coefficients and Pearson correlation coefficients. Data analysis was conducted using SPSS software, version 18.

## Results

### Participants

In the present study, 400 widows and orphans completed the diagnostic interview (widows: *n *= 194; 48.5%, orphans: *n *= 206, 51.5%). Eighteen subjects who were approached rejected participation in the trial and three subjects did not finish the interview. The sample consisted of 351 women (87.7%) and 49 men (12.3%). The participants mean age was 37.18 years (*SD *= 16.73, range 18-97 years). Education level attained varied widely with a range of 0 to 18 years of school completed (*M *= 4.93, *SD *= 3.50). The highest degree of school education was primary school for 37.0% (*n *= 148), secondary school for 4.8% (*n *= 19), apprenticeship for 5.5% (*n *= 22), university for 0.3% (*n *= 1) and 52.5% (*n *= 210) were without any school degree. The widows and orphans were Catholic (61.0%, *n *= 244), Protestant (*n *= 23.3%, *n *= 93), Islamic (4.0%, *n *= 16), Adventist (2.0%, *n *= 8), of other religion (6.0%, *n *= 24) or indicated that they were not practicing any religion (3.8%, *n *= 15).

#### Prolonged grief reactions and loss experiences

There were no significant differences in the PGD-group and the group without PGD in any of the demographic variables.

8.0% (*n *= 32) of the interviewed sample met criteria for PGD (widows: 8.8%, *n *= 17; orphans: 7.3%, *n *= 15). The majority of the sample had experienced the death of the mother (72.9%, *n *= 291), the father (90.7%, *n *= 361), at least one sibling (86.0%, *n *= 344), at least one other family member (96.8%, *n *= 387), or others (79.5%, *n *= 318). About half of the sample (48.5%, *n *= 194) had experienced the death of a partner and over a third (38.8%, *n *= 155) had lost at least one child. The mean of the types of losses experienced was 5.13 (*SD *= 1.33; range: 1-7). The majority of the interviewed orphans (61%, *n *= 125) were full orphans. There was a significant difference in grief-severity between those orphans who had lost both parents compared to those who had lost one parent, *t*(203) = - 3.48, *p *< .001, *M *= 14.76, *SD *= 8.87; *M *= 10.41, *SD *= 8.49. Of those who experienced the respective bereavement, the most distressing loss ever experienced was the partner for 57.2% (*n *= 111), the mother for 31.6% (*n *= 92), the father for 23.8% (*n *= 86), a child for 25.2% (*n *= 39), a sibling for 13.7% (*n *= 47), another family member for 5.4% (*n *= 21), and another person for 1.3% (*n *= 4). The mean age when they had experienced their worst loss was 25.72 years (*SD *= 16.52, range = 2-85). The mean time since the death associated with prolonged grief reactions was 11.50 years (*SD *= 4.15, range = 1-38). The primary cause of the prolonged grief related death was the genocide (62.0%, *n *= 246), followed by illness (27.5%, *n *= 109), accident (3%, *n *= 12) or age (1%, *n *= 4). The remaining 6.5% (*n *= 26) of the sample indicated the cause of death as "other" which was mostly poisoning and 0.8% (*n *= 3) did not know the reason of the death. The majority (70.0%, *n *= 278) had lost a loved one through violent death (during the genocide: 88.5%, *n *= 246, accident: 4.3%, *n *= 12, poisoning: 7.2%, *n *= 20). Almost half (47.6%, *n *= 117) of the participants who experienced their most distressing loss during the genocide indicated that the dead body was never retrieved. No group difference in grief severity was found between those who indicated that the dead body had been found and those who reported that the body has not been retrievable.

The majority of the respondents (68.5%, *n *= 265) indicated that a funeral ceremony for the grief related loss had taken place and that they had participated in it (*n *= 224). However, 44.0% (*n *= 176) did not or could not attend the funeral; either because no funeral ceremony had been possible or they had not been present at the ceremony.

**Figure 1:**
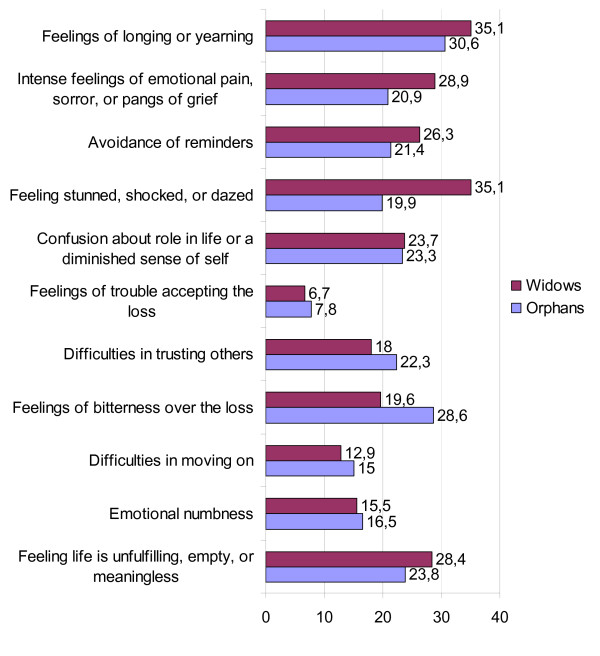
**Percentage of Prolonged Grief Disorder symptoms according to the PG-13 **[1,25]** in bereaved Rwandan widows (*n *= 200) and orphans (*n *= 194).**

The mean of the grief-score of the total sample was *M *= 24.43 (*SD *= 8.77, range: 11-53). Figure 1 reports the frequency of the PGD symptoms for orphans and widows. Comparisons between orphans and widows on the different grief measures found significant group differences for the symptoms "feeling stunned, shocked or dazed by the loss", χ^2^(1, *N *= 400) = 12.57, *p *< .001 and "feeling bitter over the loss", χ^2^(1, *N *= 400) = 4.46, *p *< .05. Whereas widows tended to be more stunned or shocked by the loss than orphans (35.1%, *n *= 68 versus 19.9%, *n *= 41), orphaned participants reported more often symptoms of bitterness than widowed subjects (28.6%, *n *= 59 versus 19.6%, *n *= 38).

Criterion B (at least one symptom of separation distress) was met by 38.0% (*n *= 152) of the sample, 12.3% (*n *= 49) fulfilled criterion C (at least 5 cognitive, emotional or behavioral symptoms), 80.5% (*n *= 322) met criterion D (symptoms have been present for at least 6 months) and 57.5% (*n *= 230) fulfilled the functional impairment criterion (criterion E). The separation distress criterion was significantly more often met by widows compared to orphans, χ^2^(1, *N *= 400) = 4.49, *p *< .05, 43.3%, *n *= 84, 33%, *n *= 68, respectively. No significant differences were found for any of the other grief variables.

#### Correlates of PGD

The results of the regression analysis are presented in table 1. Survivors with the highest grief-scores were those who had lost a loved one through violent death, had high levels of posttraumatic stress symptoms, had only recently lost someone, and whose religious/spiritual beliefs did not play an important role in their everyday life. The beta-weights indicate that the severity of symptoms of PTSD was the variable that had the highest correlation with grief severity. In addition, PGD was significantly related to the time since the loss. The violent nature of the death was found to influence the grief severity. Survivors who lost someone to violent death had more severe grief symptoms compared to participants who lost a significant other to non violent death, *M *= 26.20 (*SD *= 8.60) versus *M *= 20.14 (*SD *= 7.68). A marginal significant group difference was found between violent death due to genocide, to an accident or to poisoning, χ^2^(2, *n *= 278) = 5.18; *p *= .08. Participants who had lost someone to genocide, to an accident or to poisoning displayed an average grief score of *M *= 26.60 (*SD *= 8.78), *M *= 22.0 (*SD *= 4.02), *M *= 23.80 (*SD *= 7.52), respectively. Religious/spiritual importance appeared to be protective for the development of prolonged grief reactions. Gender, age at the time of bereavement, bereavement status (widow versus orphan), the number of types of losses, funeral participation and the control variable of age did not significantly contribute to the prediction of the severity of prolonged grief reactions. The explained variance of the model was 53.8%.

**Table 1:** Multiple Regression analyses with grief score as the dependent variable (*N *= 400)

Predictors	B_PGD-score_	B SE_PGD-score_	b_PGD-score_
Gender (female^0^/male^1^)	- .49	.99	- .02

Age at the time of bereavement	- .02	.04	- .04

Bereavement status (orphan^0^/widow^1^)	.11	1.20	.01

Mode of death (nonviolent^0^/violent^1 ^death)	2.21**	.82	.12

PTSD severity-score	.63***	.03	.69

Years from the loss	- .29***	.09	- .14

Funeral (not attending funeral^0^/attending funeral^1^)	- 1.14	.68	- .06

Number of experienced types of losses	- .04	.31	- .01

Importance of religious/spiritual beliefs	- 1.19**	.44	- .10

Note: *p < .05, ** *p *< .01, *** *p *< .001. ^0^: coded 0, ^1^: coded 1, R^2 ^of the model is .538.

## Discussion

This study investigated the bereavement history and the grief reactions among Rwandan widows and orphans, using the diagnostic criteria proposed by Prigerson and colleagues in 2008 [26]. Results indicate that a significant portion of the sample met criteria of PGD. The rather unique data set in terms of potential factors contributing to the emergence of PGD allowed us to examine the associations between PGD and various individual and contextual variables. Risk factors associated with PGD included loss to violent circumstances, PTSD symptom severity, years passed since the loss and importance of religious/spiritual beliefs.

In the present study we interviewed Rwandan orphans and widows, to ensure at least one loss experience. However, most had faced multiple types of losses, including the loss of a partner, the mother, the father, a sibling, a child, another family member or another close person with a total mean of five different types of experienced losses. Concerning the mode of the grief related death, the primary causes were genocide (62%) and illness (28%). In addition, almost half (48%) of the participants who experienced their most distressing loss during the genocide indicated that the dead body was not retrievable.

We found that a significant portion of the interviewed persons suffered from PGD at the time of the interview. The overall prevalence of PGD was 8% with a mean of 12 years after the grief-related loss. Intensive longing or yearning for the lost person was the most often reported symptom for both widows and orphans. This is congruent with the results of other studies which demonstrated that yearning for the deceased was the most commonly reported PGD symptom [8,7]. In addition, yearning had been found to constitute the core of PGD [28,29].

A number of studies have investigated prolonged grief reactions in different bereaved populations and reported PGD rates ranging from 12% to 64% [8,9,14,28,30-32]. Pivar and Field [33] found in their study with Vietnam veterans that a significant proportion displayed prolonged grief reactions due to interpersonal losses that occurred over 30 years ago. There is also evidence that those who suffer multiple losses close together grieve for greater lengths of time [34]. However, it seems difficult to compare the reported findings of prevalence rates since researchers used different diagnostic criteria for grief outcomes and most studied pathological grief reactions after a relatively short period post-loss. Furthermore, most grief research investigated responses to a single loss experience in distinct bereavement groups living in industrialized societies. In contrast, almost our entire sample has faced multiple losses making it difficult to cluster them to one bereavement group. Our results suggest that a significant proportion of the interviewed sample continues to experience grief over interpersonal losses that occurred on average 12 years ago and attest that unresolved grief will endure over time if not addressed by clinical intervention in a significant proportion of persons.

As a second goal, we examined risk factors for PGD. Regression analyses showed that individuals who experienced a loved one's death as violent, those who reported high levels of symptoms of PTSD, those who had only recently lost someone, and those participants who indicated no importance in religious/spiritual beliefs in their actual life were those participants who were more likely to display severe grief reactions. In contrast, the variables of gender, age at the time of bereavement, bereavement status, number of types of losses and the participation in a funeral service did not impact grief severity.

In the present study, female gender and age at the time of bereavement was not associated with more severe grief reactions. As a result of our sampling method to include widows (only female gender) and orphans (females and males), the majority of participants were females (88%). However, no gender differences were detected when examining group differences in orphans and half-orphans only.

Even though the majority of the sample in our study indicated the loss of a partner as the most distressing loss experience, the bereavement status as a widow did not predict the severity of prolonged grief reactions. This finding contradicts other research which found that widowhood was consistently associated with prolonged grief reactions [9,12,13]. On the other hand, the results have documented that symptoms of prolonged grief were not influenced by kinship to the deceased [16]. Our study implies that both groups - widows and orphans - are comparably affected by symptoms of prolonged grief disorder.

Few studies have examined the associations between grief reactions and the number of bereavement experiences. The multiple loss experiences reported by our study sample enabled us to examine a possible "dose-response-effect" as has been documented in the trauma literature for the development of PTSD [for example 3]. However, a "bereavement overload" in the sense that the number of reported types of loss experiences predicts grief severity did not appear in the present study. It is possible that the attachment or bonding to one single lost person might be more important than the total number of losses faced. Our results are therefore in accordance with those of Cherney and Verhey [35] who found no significant relationship between the number of individual losses reported and the intensity of grief reactions. The authors conclude that a process of habituation as an adaptive response to bereavement overload may be occurring in individuals who have faced multiple losses.

In the present study, we found that funeral attendance was not protective for the development of prolonged grief reactions. This missing association between funeral attendance and grief symptoms has been reported by other researchers [20,21]. In the present study the lack of funeral participation implied either that no funeral ever took place or that the subjects had not been present in spite of a ceremony. However, the vast majority had participated if given the opportunity. We did not collect information about the personal reason for non participation. It might be possible that those who refuse to participate when given the opportunity may have an increased risk for developing PGD. If true, this would mean that counselling should not attempt to convince a client to participate in a ritual but rather examine the reasons why a client is not interested in a ceremony.

In the present study the majority (70%) indicated that the grief-related loss had occurred through violent death. In line with existing research [12,13,36], the violent nature of the death was found to increase the risk for prolonged grief reactions. A component common to violent death includes the factor of suddenness [37], which had been found to be significantly associated with PGD [31]. According to Morgan et al. [15], persons who lose significant others to violent and unexpected death can be expected to have more difficulty grieving the loss because of the sudden disruption to their lives and the painful emotions, such as anger and guilt that are typically felt following traumatic loss. Studies suggest that PGD that follows violent loss is conceptualized as stemming from one's inability to make sense of the experience [31,37]. In addition, there is evidence that the feeling that others are accountable for the death is associated with higher PGD-scores compared with those who did not have this feeling [20], a fact which might be particularly relevant in the context of violence.

Results of the present study demonstrate that the severity of PTSD is associated with the severity of PGD. Both, PGD and PTSD may be the result of traumatic loss and may be overlapping constructs when a violent loss occurs to an attachment figure. It is also possible that symptoms of PTSD might interfere with the survivor's ability to successfully complete the mourning process. Any thoughts about the deceased may be suppressed as they may automatically trigger trauma reminders. It could be that the treatment of PTSD might facilitate the mourning process. PTSD can be successfully treated, also among traumatized survivors of the genocide [6]. It remains to be investigated if PGD-symptoms respond to similar interventions and parallel the relief from PTSD and/or depression symptoms.

Most studies examined differences in grief reactions within a relatively short period after the loss and found that time since the loss did not significantly impact the severity of prolonged grief symptoms or PGD diagnosis [9,12,16,17]. The focus on a sample of bereaved widows and orphans who considerably ranged in length of time since the death enabled the evaluation of the predictive power across a considerable period of time. We found that time since the loss (measured in years) was significantly associated with the severity of grief reactions. This implicates that distressing and painful grief reactions might decrease as time goes by. Our results are in accordance with Keesee et al. [36] who examined a sample of bereaved parents over a period of five years post-loss and found that the length of bereavement uniquely contributed to the intensity of grief symptoms.

The results of the present study confirm the findings from other studies where religious/spiritual belief has appeared to be protective against problematic grief affect [22]. This belief system might offer potential consolation and the knowledge that there will be an afterlife and a reunification of family members might have helped them through bereavement. It is also possible that the loss experience increased the importance of their religious beliefs, as has been shown in a longitudinal study by Brown and colleagues [25]. This increase in turn has been found to be associated with decreased grief reaction. On the other hand, religious belief might help them to find meaning in the loss, a factor that has been found to be associated with grief reactions by numerous studies [31,36]. Future research needs to deeper understand the ways in which religious belief is helpful.

Our study has several limitations. Due to the cross-sectional and retrospective nature of the design, it is impossible to establish causal or temporal relationships between the different variables. The sample consists of individuals who had faced multiple losses with the majority of losses due to violence. However, we did not distinguish if the different losses had occurred within a short period of time or if losses occurred repeatedly over the years. The focus of the present study was on women (who outnumbered men approx. 7:1). The evaluation has been based exclusively on subjective assessment by the bereaved themselves.

## Conclusions

To our knowledge this is the first study that contains such detailed information about loss experiences and grief reactions in Rwandan genocide survivors. The data demonstrate that PGD occurs in a significant portion of survivors, even many years post-loss and that the severity of grief reactions may be associated with a set of distinct risk factors. Subjects who lose someone through violent death seem to be at special risk as they have to deal with the loss experience as such and the traumatic aspects of the loss. Symptoms of PTSD may hinder the mourning process and may need to be addressed first, before the mourning process can be completed. Religious/spiritual belief appeared to be protective against PGD as it may help to better accept and to find meaning in the loss. These aspects need to be considered in the treatment of PGD.

## Competing interests

The authors declare that they have no competing interests.

## Authors' contributions

SS conceived of the study, participated in its design and the coordination of the study, participated in assessments, performed the statistical analyses and drafted the manuscript. NJ conceived of the study, participated in the design, the coordination and the assessments of the study. JPD participated in the design and the coordination of the study. TE participated in the design of the study and contributed to the interpretation of findings and writing of the paper. All authors read and approved the final version.

## Pre-publication history

The pre-publication history for this paper can be accessed here:

http://www.biomedcentral.com/1471-244X/10/55/prepub
